# Metals Bioaccumulation in 15 Commonly Consumed Fishes from the Lower Meghna River and Adjacent Areas of Bangladesh and Associated Human Health Hazards

**DOI:** 10.3390/toxics10030139

**Published:** 2022-03-12

**Authors:** Mohammad Belal Hossain, Fatema Tanjin, M. Safiur Rahman, Jimmy Yu, Shirin Akhter, Md Abu Noman, Jun Sun

**Affiliations:** 1 Department of Fisheries and Marine Science, Noakhali Science and Technology University, Noakhali 3814, Bangladesh; tanjin.20151151@gmail.com; 2 School of Engineering and Built Environment, Griffith University, Brisbane, QLD 4111, Australia; jimmy.yu@griffith.edu.au; 3 Atmospheric and Environmental Chemistry Laboratory, Chemistry Division, Atomic Energy Centre Dhaka (AECD), Dhaka 1000, Bangladesh; safiur.rahman@baec.gov.bd (M.S.R.); shirinakhter43@yahoo.com (S.A.); 4 State Key Laboratory of Biogeology and Environmental Geology, China University of Geosciences, Wuhan 430074, China; abu.noman.nstu@gmail.com

**Keywords:** heavy metals, emerging pollutants, human health hazard, carcinogenic risks, fish market, tropical estuary

## Abstract

The lower Meghna River, the easternmost part of the Ganges Delta, faces severe anthropogenic perturbations as it receives a huge discharge and industrial effluents. To measure the metal concentrations and human health hazards, edible tissues of 15 commercially important fish species were collected from the local fish markets and the lower Meghna River, Bangladesh. Trace and heavy metals such as Pb, Cr, Cu, Zn, Mn, Fe, Hg, Ni, Ca, Co, Se, Rb, Sr, and As were detected using the Energy Dispersive X-ray Fluorescence (EDXRF) method. The hierarchy of mean metal concentrations obtained was: Fe (162.198 mg/kg) > Zn (113.326 mg/kg) > Ca (87.828 mg/kg) > Sr (75.139 mg/kg) > Cu (36.438 mg/kg) > Se (9.087 mg/kg) > Cr (7.336 mg/kg) > Mn (6.637 mg/kg) > Co (3.474 mg/kg) > Rb (1.912 mg/kg) > Hg (1.657 mg/kg) > Ni (1.467 mg/kg) > Pb (0.521 mg/kg) > As (BDL). Based on the metal concentration obtained, the carnivorous species contained more metals than omnivores and herbivores. Similarly, the euryhaline and benthic feeder fishes had more metals than the stenohalines and demersal fishes. The metal pollution index (MPI) suggested that the highly consumed fish species Tilapia (*Oreochromis mossambicus*) and Rui (*Labeo rohita*) accumulated higher metals than other fishes. Both the Targeted Hazard Quotient (THQ) and Hazard Index (HI) values for adult and child consumers were <1, indicating that consumers would not experience the non-carcinogenic health effects. Although children were more susceptible than adults, carcinogenic risk (CR) exposure of Cr for all the consumers was found in the acceptable range (10^−6^ to 10^−4^), but the CR exposure of Pb was negligible for all the consumers. The correlation, principal component analysis (PCA), and cluster analysis were conducted to identify the sources of metals identified from the fish tissue. The results indicated that the probable sources of the pollutants were anthropogenic, arising from agricultural activities, electroplating materials, and lubricants used near the study area. However, the present study showed a different metal concentration in the samples at different levels but within the threshold levels non-carcinogenic and carcinogenic health risks; hence, the fishes of the area, in general, are safe for human consumption.

## 1. Introduction

In recent decades, extensive population growth and subsequent economic development have aroused a global consciousness about heavy metals contamination owing to their persistence, non-biodegradability, and stability [1,2]. Heavy metal refers to any metallic chemical element with a relatively high density (more than 5 gm/cm^3^) and which is toxic or poisonous at low concentrations [3]. Some of those elements (e.g., Zn and Cu) play a vital role in biological systems, but they are toxic at elevated levels. However, some of these elements are noxious even at low levels (e.g., Hg, Ni, Cr, Pb, and Cd), and are therefore categorized as potentially harmful elements [4,5]. Those toxic metals naturally occur in aquatic environments in deficient concentrations, but their concentration levels have increased due to anthropogenic activities over time [6]. Heavy metals may enter aquatic ecosystems via atmospheric deposition, agricultural, industrial, and domestic activities [7]. Hence, water bodies like lake, rivers, and estuaries has been identified as a key reservoir of heavy metals due to the center of urbanization and industrialization [1,8]. Those metals discharged into the aquatic environment [9] can affect the organisms and ecosystem due to their toxicity, long persistence, and accumulative behavior [10,11], and finally assimilated by human consumers, resulting in health risks. However, due to a poor waste management and disposal strategy, the concern is growing more seriously worldwide, and the intensity is higher in the developing countries [12,13,14].

Among the aquatic organisms, fish is a major constituent of a well-balanced diet with a healthy energy source offering low cholesterol levels, high-quality proteins, omega-3 fatty acids, vitamins, and other vital nutrients [15,16]. Therefore, fish consumption has increased worldwide in recent years, particularly with the awareness of its nutritional and therapeutic benefits. For example, the American Heart Association recommended consuming fish at least twice per week to reach the daily intake of omega-3 fatty acids [17]. However, there is a concern that heavy metals accumulated in edible fish may represent a health risk, especially for populations with high fish consumption rates [18]. The presence of toxic heavy metals in fish can invalidate their beneficial effects. However, fish typically accumulate heavy metals from food, water, and sediments [19,20]. Therefore, fish are good indicators of the heavy metal contamination levels in aquatic systems [21], because the metal levels in fish usually reflect the levels found in sediment and water of the particular aquatic environment from which they are sourced [22], and time of exposure [23]. As the anthropogenic activities are effortlessly generating heavy metals in sediment and water, and pollute the aquatic environment [24], many local and international monitoring programs have been established to assess the quality of fish for human consumption and monitor the health of the aquatic ecosystem [25]. Because diet is the main route of exposure to metals, hence, the risk assessment of these elements to humans via dietary intake is essential [26].

The lower Meghna River acts as one of the potential sources of different biological species and economic trends, where countryside people are directly or indirectly involved in commercial fishing activities and catches. For instance, *Tenualosa ilisha* 2.72 kg/h/100 m is caught using a gill net alone in the river estuary [27]. Moreover, around 3500 tons of the other selective fish species are captured per year, potentially contributing to the fish supply in the commercial fish markets and the country’s total fish production. However, the estuary engulfs huge volumes of noxious wastes discharged from thousands of industrial units and sewerage lines which make it one of the most polluted estuarine systems in Bangladesh [28]. The discharge of untreated industrial materials, unused battery particles, painting materials derived from Pb sources and discharged gasoline from cargos, transportation routes for launch-steamer and mechanized boat, and unsuitable domestic discharged wastage provides a comprehensive source of heavy metals in the Meghna River [29].

Noakhali is a coastal district of Bangladesh where people mostly consume fish mainly from the Meghna River Estuary and some aquaculture farms. Fishes from the Meghna River Estuary are transported to different fish markets of Noakhali. Therefore, the analysis of heavy metals in fishes from this area is indispensable as a major human health concern. Numerous studies have been published on heavy metals in the aquatic environment of Bangladesh [30], such as the Buriganga river [31], Bangshi river ([32], Meghna river [28], Dhaleshwari river [29]. However, there is limited knowledge on the concentration of heavy metals in the most consumed fishes from Noakhali Fish Market and their potential health risks through their consumption. In addition, in the coastal rural area of Bangladesh, the general people and authorities are unaware of the health implication of heavy metals accumulation in fish. Therefore, this study aims to determine the heavy metal concentration in fish from the Noakhali fish market and evaluate the risk of heavy metals to human health, which will provide insights to local people and management authorities to take necessary steps regarding this issue.

## 2. Materials and Methods

### 2.1. Sample Collection, Preparation and Analysis

**Figure 1:**
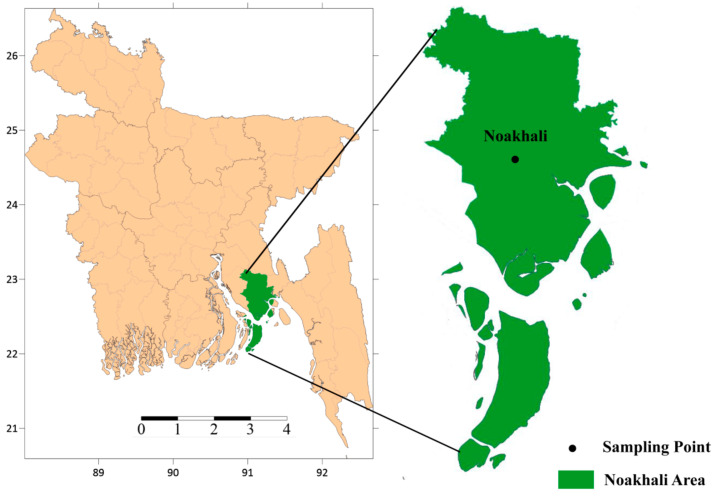
Fish sampling point in Noakhali and adjacent areas.

A total of 30 fish samples of 15 species (two samples per species) from different feeding habitats (Table 1) were collected from different fish markets of Noakhali and the lower Meghna River to evaluate heavy metal levels (Figure 1). These species were selected because these are the mostly consumed throughout the country, available at almost all the fish markets and commercially important. After collection, all the fish samples were kept frozen (–20 °C) by storing them in an icebox and transported to the laboratory as soon as possible. After measuring the physical parameters (weight and length), each fish sample was cleaned and washed with deionized water. Then, it was chopped with a stainless-steel knife cleaned with acetone and hot distilled water before use. Fourteen trace and heavy metals (Pb, Cr, Cu, Zn, Mn, Fe, Hg, Ni, Ca, Co, Se, Rb, Sr, and As) were analyzed from the dissected fish muscle tissue. For analyzing the metals, fish flesh was taken into a beaker and kept in a muffle furnace at 300 °C for 3 h to make ash. The ash samples were ground for making powder using carbide mortar and pestle. For EDXRF analysis, each powdered piece was pressed into a pellet of 2.5 cm diameter with a hydraulic press pellet maker (Specac) using 7 (seven) tons pressure. The irradiation of all actual samples was performed by assigning a time-based program controlled by a software package provided with the EDXRF system. The standard materials were also irradiated under similar experimental conditions to construct the calibration curves for quantitative elemental determination in the respective samples. The generated X-ray spectra of the materials were stored in the computer.

### 2.2. Analytical Quality Control

Before using, all the glassware and plastics were washed in nitric acid solution for 15 min and then rinsed with deionized water. Analytical grade reagents (Merck; Germany) and de-ionized water were used for the analysis throughout the study. For the validation and accuracy, the analytical method certified reference material (CRM 320, Merck KGaA, Darmstadt, Germany) was used. The recovery rate of the of the selected metals in the applied analytical method ranged from 72% to 105%. Besides, during the analytical process, the contamination’s influence was absent and the relative standard deviation (RSD) was ≤10% for all tests.

### 2.3. Human Health Risk Assessment of Heavy Metals

#### 2.3.1. Metal Pollution Index (MPI)

To assess the metal pollution, the metal pollution index (MPI) was adopted as follows [33,34]:

MPI = (CM_1_ × CM_2_ × CM_3_ × …× CM*_n_*)^1/*n*^

where CM_1_ is the concentration of the first concerning metal, CM_2_ is the concentration of the second concerning metal, CM_3_ is the concentration of the third concerning metal, CM*_n_* is the concentration of the nth metal (mg/kg dry wt) in the tissue sample of a particular species.

#### 2.3.2. Estimated Daily Intake (EDI)

Estimated daily intake (EDI) was calculated by the following equation [35,36]:

EDI = (Cn × IGr)/Bwt

where Cn is the concentration of metal in the selected fish muscles tissue (mg/kg dry wt); IGr is the acceptable ingestion rate, which is 55.5 g/day for adults and 52.5 g/day for children [37,38]; Bwt is the bodyweight: 70 kg for adults and 15 kg for children [37].

#### 2.3.3. Target Hazard Quotient (THQ) for Non-Carcinogenic Risk Assessment

THQ was estimated by the ratio of EDI and oral reference dose (RfD). The ratio value <1 implies non-significant risk effects [39]. The THQ formula is expressed as follows [40,41].

$$\mathrm{THQs}=\frac{\mathrm{Ed}\times \mathrm{Ep}\times \mathrm{EDI}}{\mathrm{At}\times \mathrm{RfD}}\;\times \;10^{-3}$$

where Ed is exposure duration (65 years) [37]; Ep is exposure frequency (365 days/year) [31]; At is the average time for the non-carcinogenic element (Ed × Ep).

#### 2.3.4. Hazard Index (HI)

Hazard index (HI) was calculated for the multiple elements (Hg, As, Mn, and Cr) found in the fish samples, and the equation is as follows [8].

$$\mathrm{HI}\;=\overset{n}{\underset{i\;=\;k}{\sum }}\mathrm{THQ}$$

where THQ are the estimated risk value for individual metal [8]. When the HI value is higher than 10, the non-carcinogenic risk effect is considered high for exposed consumers [42,43].

#### 2.3.5. Carcinogenic Risk (CR)

To assess the probability of developing cancer over a lifetime, the carcinogenic risk is evaluated for the consequence of exposure to the substantial carcinogens [44,45]. The acceptable range of the risk limit is 10^−6^ to 10^−4^ [46,47]. CRs higher than 10^−4^ are likely to increase the probability of carcinogenic risk effect [48,49]. The established equation to assess the CR is as follows [38,41].

$$\mathrm{CR}=\frac{\mathrm{Ed}\times \mathrm{Ep}\times \mathrm{EDI}\times \mathrm{CSF}}{\mathrm{AT}}\;\;\times \;10^{-3}$$

where CSF is the oral slope factor of a particular carcinogen (mg/kg-day) [47].

### 2.4. Statistical Analysis

The correlation matrix (CM), principal component analysis (PCA), and Hierarchical cluster analysis were performed through PAST (version 3). CM and PCA help to determine the correlation between heavy metals in fish tissue [50]. Hierarchical cluster analysis is one of the most widely used hierarchical algorithms, which results in clusters in which variables or individuals are added in sequence considering the hierarchy of the cluster [35]. Clustering of metals concentrations in fish muscles based on Bray–Curtis similarity was performed to plot elements in a separate cluster, thus differentiating the samples’ contamination status [51].

## 3. Results and Discussion

### 3.1. The Concentration of Heavy Metals in Fish Muscle Tissue

Heavy metal contamination in fish is one of the severe threats to humans and aquatic animals. Determination of heavy metal concentration is the first step to evaluating the extent of pollution in fish. The concentration (wet weight) of selected metals was in the following descending order: Fe (162.198) > Zn (113.326) > Cu (36.438) > Cr (7.336) > Mn (6.637) > Hg (1.657) > Ni (1.467) > Pb (0.521) (Table 2). Among the examined fishes, the average metal concentration was maximum in Tilapia and maintained the following descending trend: Tilapia > Rui > Gulia > Poa > Loitta > Chiring > Ricksha > Bata > Catla > Grass Carp > Kalibaus > Koral > Pabda > Koi > Big head carp. However, considering the feeding guild of the sampled fishes, carnivorous species had the highest metal concentrations followed by herbivores and omnivores. Besides, the euryhaline fishes possessed higher metal concentrations then the stenohaline fishes. Moreover, the average concentration of metals in demersal fishes was lower than the benthic fishes. However, the concentrations of metals in sampled fishes differed largely, which might be a result of different ecological needs, metabolism, and feeding patterns of the examined fishes [52,53]. Many studies reported the metal concentrations in fishes depend mostly on their habitat type [52,54]. It is commonly observed that the sediment is the major uptake pathway for metal contamination and plays a critical role in the heavy metal uptake for fish [55]. Fish living near the sediments of the waterbody and feeding on humic substances and benthic invertebrates accumulate and transfer heavy metals from sediments to fishes [55]. Therefore, benthic and benthopelagic fishes generally exhibit higher concentration of metals than demersal fishes [36,54]. Besides, a previous study reported that the metal concentrations in piscivorous species in the higher trophic level tend to accumulate more metals than omnivorous and herbivorous species [55], which supports our findings. However, this finding suggests that the metal concentrations in fishes are not only influenced by the habitat but also bio-accumulation through the food chain [14,55,56,57,58].

In the present study, the mean concentration of Cu in the fish was 36.44 ± 9.19 (mg/kg wet weight). The concentration of Cu was found to vary from 30.29 ± 3.934 to 48.59 ± 9.935 (mg/kg) among all the fishes. The highest concentration of Cu was found in Grass carp (48.59 ± 9.935 mg/kg), whereas the lowest concentration was found in Poa (30.29 ± 3.934 mg/kg). However, the Cu concentration was higher compared to the national and international guideline values and the previous studies of the same geographic region (Table 3). Previously, the maximum concentration of Cu in Bangladesh was recovered from the Bangshi river [32]. Even most of the international guidelines restricted the Cu concentrations within 30 mg/kg. The concentration of Cu we obtained surpassed all the previous findings in Bangladesh and other international guidelines as well (Table 3). Besides, the range of Cu concentration found in the fishes from Asafo market, Ghana, ranged between 0.02–0.156 [59], and Pearl river, China, was within 1.17–6.72 [60]. All these studies reported lower concentration of Cu than our findings. Though the optimum concentration of Cu is important for the body as it produces hemoglobin and some other vital enzymes, the excess amount may lead to malfunction of liver and kidney [38]. Notably, for the trace element Cu, the maximum recommended level for 1–3 years old children is 1.0 mg/day, and for 19–70 years old males/females, it is 10 mg/day. Therefore, an excess amount of Cu over the recommended levels may lead to organ damage (kidney, liver) [61].

In terms of Pb, the concentrations in fish muscles ranged from 0.202 to 0.68 (mg/kg wet weight). The highest Pb concentration was found in Koi (0.68 mg/kg), whereas the lowest Pb concentration was found in Loitta (0.202 mg/kg). Pb concentrations found in the examined fishes maintained the following decreasing order: Koi > Chiring > Bata > Bighead carp > Rui > Catla > Gulia > Kalibaus > Grass carp > Pabda > Ricksha > Koral > Tilapia > Poa > Loitta. Based on the FAO [62] and WHO [77], maximum permissible concentrations for Pb are 2 and 0.5 mg/kg, respectively. Based on the concentrations obtained, Pb concentrations in the muscle of all fishes were below the threshold limit from the WHO [77]. However, the mean Pb concentrations in the present study were lower than the data reported earlier for the Koral and Poa in the same geographic region [75]. Besides, a more or less similar Pb concentration were obtained in fish tissue by Staniskiene et al. [78] and Copat et al. [79].

Optimum Cr concentration in the diet has an important role in lipid and glucose metabolism [31,80]. However, the excess Cr consumption may lead to acute pulmonary disorders and organ damage like lungs, kidney, and liver [18,81]. The recommended maximum permissible concentration for Cr is 50 mg/kg from the WHO [77]. In our study, the mean Cr concentration in the muscle of fish ranged from BDL to 9.685 mg/kg and did not exceed the proposed limit from the WHO [77]. Cr concentrations among the fish species maintained the following descending order: Poa > Koral > Koi > Grass carp > Chiring > Ricksha > Bata > Bighead carp. However, the mean Cr concentration in the muscle tissues of Poa, Koral, Koi, Grass carp, Chiring, Ricksha, Bata, and Bighead carp in the present study was found to be higher than the data reported for eight species from the Meghna river estuary [75] and was found to be lower than the concentration found in *C. carpio* and *S. lucioperca* in the Beysehir Lake [82].

The Fe concentration was the maximum obtained compared to all other elements analyzed in the different species of fishes. The maximum permissible concentration for Fe is 100 mg/kg [83]. In the present study, the mean concentration of Fe was 162.198 mg/kg, and ranged largely among the species. The highest concentration of Fe recorded in Bighead carp (208.51 mg/kg) and the lowest value was in Koral (135.58 mg/kg). Fe concentrations in the muscles of fifteen fish species were in the following decreasing sequence: Bighead carp > Grass carp > Ricksha > Poa > Catla > Bata > Chiring > Gulia > Tilapia > Kalibaus > Loitta > Pabda > Rui > Koi > Koral. Based on the values recovered, Fe concentrations found in the muscles of all fishes exceeded the permissible limit by the WHO [83]. Besides, the concentration of Fe was higher than the earlier study of Bhuyan et al. [28], where the range of Fe reported was 7.85 to 147.77 mg/kg. However, mean concentrations of Fe in all species were lower than fishes from Gorgan Bay [76]. Fe is an essential micronutrient for the fishes, as a vital component regarding cellular respiration and oxygen transfer [84]. However, acute Fe overdose is potentially life threatening and also slowly developing damages to organs like heart and liver [18]. Besides, the excess amount of Fe acts as a catalyst in Fenton reaction, responsible for generating free radicals which is toxic [85].

In the present study, the mean Zn concentrations in the muscle of fish species were 113.326 mg/kg. Zn concentrations were found in fish in the following sequence: Bata > Rui > Gulia > Tilapia > Grass carp > Chiring > Kalibaus > Poa > Koi > Loitta > Catla > Koral > Bighead carp > Pabda > Ricksha. According to the FAO/WHO [86], the maximum permissible amount of Zn for human consumption is 30 mg/kg. Zn concentrations found in the muscles of all fishes exceeded the guideline value [86]. The concentration level of Zn in the fishes is almost alike to the reported value of the Bangshi river [32]. However, the mean concentrations of Zn in all species were higher than the other international reports [60,79,87,88]. Zn has a tendency to be accumulated in the fatty tissues of fishes and other aquatic organisms, and likely to affect the reproductive physiology in fishes [89]. Besides, the chronic exposure to Cu and Zn is reported to be associated with Parkinson’s disease [90].

According to the FAO/WHO [68], the maximum permissible concentration for Hg is 0.5 mg/kg for human. There is no known physiological requirement for Hg in animal metabolism, and high Hg exposures can result in severe toxicity [91]. In the present study, the mean Hg concentration in the muscle of fish species was 1.657 mg/kg. Hg concentrations decreased in the following order: Loitta > Catla > Koral > Poa > Ricksha > Bata > Rui > Gulia > Grass carp > Bighead carp > Tilapia > Chiring > Koi > Pabda > Kalibaus. From the hierarchy, the highest value of Hg was 2.899 mg/kg in Loitta, and the lowest value was 0.72 in Kalibaus. Hg concentrations found in muscles of all fishes were above the proposed limit by the FAO/WHO [68], which may pose a threatening consequence. However, the concentration of Hg was in line with the results of Ullah et al. [92], where the concentration range was from 0.021 to 0.121 mg/kg in the highly consumed cultured fish in Bangladesh. However, the mean concentration of Hg in all species were lower than the fish of the Pearl river and marine fish in Malaysia [60,93].

### 3.2. Metal Pollution Index (MPI)

The MPI was considered using heavy metal concentrations in the fish species and used to compare the total metal contents of the muscle of the examined fishes. The MPI is generally used to define the polluted degree of heavy metals in tissues of fish. It is considered that the higher value of estimated MPI describes the higher degree of contamination in fish [94]. The highest MPI value was obtained for Tilapia and the lowest for the Pabda. The distribution pattern of total concentrations of heavy metal accumulations in the studied fish species follow the order: Tilapia > Rui > Poa > Loitta > Ricksha > Gulia > Bighead carp > Catla > Koral > Grass carp > Koi > Kalibaus > Chiring > Bata > Pabda (Table 4). In recent years, the Tilapia has been the most consumed and cultured fish species in Bangladesh. Therefore, the high MPI value of Tilapia is a matter of metal contamination-related health hazards to local people.

### 3.3. Human Health Risk Assessment

Fish constitute a significant part of the diet of Bangladeshi people. Herein, we anticipated that the local population consumes fish and, since muscle is the most edible part of fish for humans, its intake risks must be taken into account. However, the risk assessment results are summarized in Table 4, Table 5 and Table 6 for EDI (estimated daily intake), THQ (target hazard quotient), and CR (carcinogenic risk), respectively.

#### 3.3.1. Estimated Daily Intake (EDI)

Heavy metals tend to accumulate in various organs of aquatic organisms, especially in fish, which may enter into the human metabolism through consumption, causing severe health hazards [95]. Thus, the daily intake of some selected trace metals was estimated and compared with the recommended values to assess whether the metal levels found in fish samples from the Noakhali fish market were safe for human consumption (adults & children) (Table 5). This study considered only the fish muscle, as humans mostly consume this portion. The highest recorded EDI values were 0.1285 and 0.5677 (mg/day/person) found in Fe for adults and children, whereas the lowest recorded EDI values were found in Pb (0.0004 and 0.0018 mg/day/person for adults and children). Children’s EDI values were higher than the EDI values of adults for all the metals. Ingestion of the metals through the intake of aquatic foods was the primary exposure path instead of possible risk effect from inhalation and direct dermal contact [96]. For ingestion, the results in the study area for adults and children were below the recommended daily allowance (RDA), presented in the following descending order: Fe > Zn > Cu > Cr > Mn > Hg > Ni > Pb. Therefore, EDIs, lower than RDA, indicated a possible lower health effect for the targeted groups of people (adults and children). However, it was not a permanent measurement process to conclude ‘acceptable limit’ and ‘unacceptable limit’, based on doses lower than RDA/Rfd [38,40].

**Table 5:** The EDI, RDA recorded for the different heavy metals detected in the fish species.

Elements	Mean Concentration (mg/kg)	EDI (mg/Day/Person)		Recommended Daily Dietary Allowance (mg/Day/Person)	References
		Adult	Child		
Pb	0.521	0.0004	0.0018	0.25	[63]
Cr	7.336	0.0058	0.0257	0.23	[63]
Cu	36.438	0.0289	0.1257	35	[63]
Zn	113.326	0.0899	0.3966	18–60 ^a^	[97]
Mn	6.637	0.0053	0.0232	2–5 ^b^	[98]
Fe	162.198	0.1285	0.5677	13.6	[63]
Hg	1.657	0.0013	0.0058	0.03	[68]
Ni	1.467	0.0012	0.0051	0.3 ^c^	[99]

^a^ PMTDI: provisional maximum tolerable daily intake; ^b^ ESADDI: estimated safe and adequate daily dietary intake; ^c^ Average daily intake from food.

#### 3.3.2. THQ and HI

THQ and HI proposed by USEPA [100] are the parameters for risk assessment that compare the ingestion amount of a pollutant with a standard reference dose and have been widely used in the risk assessment of metals in contaminated foods [76]. In addition, the THQ value has been recognized as one of the reasonable parameters for the risk assessment of metals associated with consuming contaminated fish [101]. The threshold limit for THQ is 1 suggested by USEPA [49]. The result described that the mean THQ of all the species was below 1 for both adults and children (Table 6). None of the metals in all the species exceeded the threshold limit, which indicates that the intakes of metals by consuming these species do not result in an appreciable hazard on the human body. The highest THQ were 8.17 × 10^−3^ and 3.61 × 10^−2^ found in Cr, and the lowest THQ were 1.03 × 10^−4^ and 4.06 × 10^−4^ found in Cu for both adults and children, respectively. For all the cases, the THQ was higher in children than adults.

**Table 6:** Non-carcinogenic (THQ) of metals for different age consumers of the targeted species of Noakhali fish market.

Species	THQ (Cr)		THQ (Fe)		THQ (Cu)		THQ (Pb)		HI	
	RfD: 0.003 *		RfD: 0.7 **		RfD: 0.3 *		RfD: 0.002 *			
	Adult	Child	Adult	Child	Adult	Child	Adult	Child	Adult	Child
Tilapia	5.75 × 10^−2^	2.54 × 10^−1^	1.69 × 10^−4^	7.46 × 10^−4^	9.55 × 10^−5^	4.22 × 10^−4^	1.68 × 10^−4^	7.44 × 10^−4^	5.80 × 10^−2^	2.56 × 10^−1^
Koi	2.08 × 10^−3^	9.17 × 10^−3^	1.66 × 10^−4^	7.34 × 10^−4^	8.59 × 10^−5^	3.79 × 10^−4^	2.69 × 10^−4^	1.19 × 10^−3^	2.6 × 10^−3^	1.15 × 10^−2^
Catla	-	-	1.91 × 10^−4^	8.44 × 10^−4^	8.96 × 10^−5^	3.96 × 10^−4^	2.26 × 10^−4^	9.97 × 10^−4^	5.06 × 10^−4^	2.24 × 10^−3^
Rui	-	-	1.67 × 10^−4^	7.38 × 10^−4^	8.85 × 10^−5^	3.79 × 10^−4^	2.40 × 10^−4^	1.06 × 10^−3^	4.95 × 10^−4^	2.17 × 10^−3^
Grass carp	2.03 × 10^−3^	8.97 × 10^−3^	2.32 × 10^−4^	1.02 × 10^−3^	1.28 × 10^−4^	1.21 × 10^−4^	2.10 × 10^−4^	9.28 × 10^−4^	2.6 × 10^−3^	1.1 × 10^−2^
Bighead carp	1.56 × 10^−3^	6.89 × 10^−3^	2.36 × 10^−4^	1.04 × 10^−3^	8.81 × 10^−5^	5.55 × 10^−4^	2.42 × 10^−4^	1.07 × 10^−3^	2.13 × 10^−3^	9.55 × 10^−3^
Kalibaus	-	-	1.69 × 10^−4^	7.46 × 10^−4^	9.98 × 10^−5^	4.41 × 10^−4^	2.12 × 10^−4^	9.37 × 10^−4^	4.81 × 10^−4^	2.12 × 10^−3^
Bata	1.58 × 10^−3^	6.99 × 10^−3^	1.77 × 10^−4^	7.83 × 10^−4^	9.40 × 10^−5^	4.15 × 10^−4^	2.51 × 10^−4^	1.11 × 10^−3^	5.22 × 10^−4^	9.29 × 10^−3^
Pabda	-	-	1.68 × 10^−4^	7.43 × 10^−4^	8.36 × 10^−5^	3.69 × 10^−4^	2.10 × 10^−4^	9.27 × 10^−4^	4.62 × 10^−4^	2.04 × 10^−3^
Poa	2.56 × 10^−3^	1.13 × 10^−2^	2.01 × 10^−4^	8.87 × 10^−4^	8.01 × 10^−5^	3.53 × 10^−4^	1.50 × 10^−4^	6.61 × 10^−4^	2.99 × 10^−3^	1.32 × 10^−2^
Chiring	1.94 × 10^−3^	8.57 × 10^−3^	1.71 × 10^−4^	7.56 × 10^−4^	2.24 × 10^−4^	5.48 × 10^−4^	2.57 × 10^−4^	1.13 × 10^−3^	2.49 × 10^−3^	1.1 × 10^−2^
Ricksha	1.91 × 10^−3^	8.44 × 10^−3^	2.13 × 10^−4^	9.41 × 10^−4^	8.97 × 10^−5^	3.96 × 10^−4^	1.90 × 10^−4^	8.41 × 10^−4^	2.41 × 10^−3^	1.12 × 10^−2^
Gulia	-	-	1.71 × 10^−4^	7.54 × 10^−4^	1.23 × 10^−4^	5.43 × 10^−4^	2.25 × 10^−4^	9.94 × 10^−4^	5.19 × 10^−4^	2.29 × 10^−3^
Loitta	-	-	1.69 × 10^−4^	7.44 × 10^−4^	9.36 × 10^−5^	4.13 × 10^−4^	8.01 × 10^−5^	3.54 × 10^−4^	1.18 × 10^−3^	1.14 × 10^−3^
Koral	2.34 × 10^−3^	1.03 × 10^−2^	1.54 × 10^−4^	6.78 × 10^−4^	8.31 × 10^−5^	3.67 × 10^−4^	1.74 × 10^−4^	7.66 × 10^−4^	2.75 × 10^−3^	1.21 × 10^−2^
Mean	8.17 × 10^−3^	3.61 × 10^−2^	1.84 × 10^−4^	8.10 × 10^−4^	1.03 × 10^−4^	4.06 × 10^−4^	2.07 × 10^−4^	9.14 × 10^−4^		

* [37] ** [100].

The findings enhanced the necessity of evaluating hazard index (HI), where surpassed HI unit expositions determined the alarming concern of health risk for the local consumers [50,102]. The investigated HI did not surpass the suggested limit. Our findings revealed that the HI of metals for species maintained the descending order: Tilapia > Poa > Koral > Koi > Grass carp > Chiring > Ricksha > Bighead carp > Loitta > Bata > Gulia > Catla > Rui > Kalibaus > Pabda. HI exceeding 1 indicates that the metals are toxic and hazardous to human health [101]. In the present study, the average HI values for all fish species were below the threshold value, which indicates that the intakes of metals by consuming those fishes do not result in an appreciable hazard risk for the human body. However, due to the absence of a definite dose relationship, THQ and HI are not considered as a direct measurement of risk concern [103].

#### 3.3.3. Carcinogenic Risk (CR) Assessment

Due to the unavailability of the carcinogenic slope factor for maximum metals, carcinogenic risk (CR) was calculated only for Pb and Cr (Table 7). The range of the CR found in the selective organisms for Pb and Cr was 1.362 × 10^−9^ to 4.583 × 10^−9^ and 2.341 × 10^−6^ to 8.629 × 10^−5^ in adults, respectively, while 6.009 × 10^−9^ − 2.023 × 10^−8^ and 1.033 × 10^−5^ − 3.809 × 10^−4^ in children. Generally, a CR value above 10^−4^ is unacceptable, whereas CR ranging from 10^−4^ to 10^−6^ is regarded as an acceptable carcinogenic risk, and below 10^−6^ is negligible [40]. In our study, CR value of Pb was negligible for both adults and children, and the CR exposures of Cr was in the acceptable range for both adults and children. The results also specified that children were more susceptible to CR exposures than adults.

### 3.4. Source Identification

The strong and moderate correlation between elements indicates their sources are similar, especially from the point and non-point sources [28]. If no correlation exists among the elements, even a single factor does not control the metals [104]. In the correlation matrix, there was a strong positive correlation between Ca vs. Sr (0.80323) with a 99% confidence level (*p* < 0.01 significance), a moderate negative correlation between Hg vs. Pb (−0.6004), and a moderate positive correlation between Fe vs. Rb (0.5551) with 95% significance level (*p* < 0.05 significance) (Table 8). Such correlation indicates that their origins are probably similar, and they might have a common anthropogenic source like industrial effluents, municipal wastes, and agricultural inputs. The study region is strongly polluted with Ca and Sr and moderately contaminated with Hg, Pb, Fe, and Rb.

**Figure 2:**
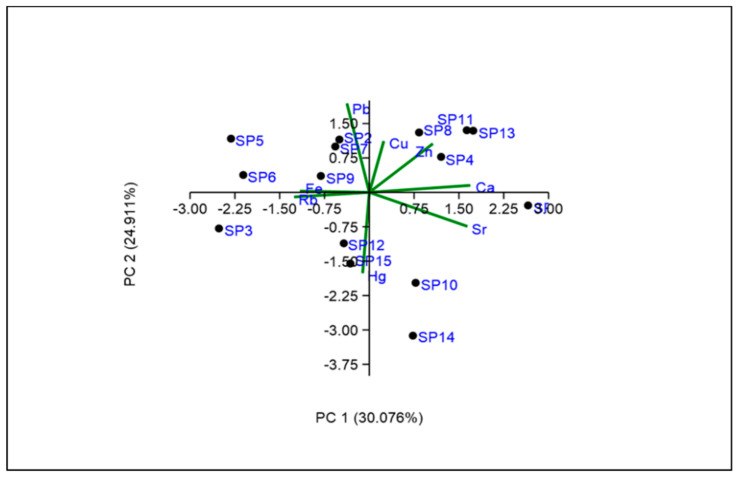
Loadings plot of rotated PCA of 8 metals in the fish sample.

In PCA, the components were taken into account whose eigenvalues were greater than 0.5 (Figure 2 and Table 9). PCA explained 99.99% of the data variation and a total of 8 significant PCs were extracted with an eigenvalue > 1. PC1 explained 30% of the total variances and exhibited an eigenvalue of 2.41. PC1 was dominated by Ca and Sr with the loadings of 0.54 and 0.52, respectively. The employed PCA revealed that the source of origin of the metal was anthropogenic. Ca, Sr, Pb, and Fe were the dominant compounds in PCA analysis due to their high loading scores in respective components. PC2 explained around 25% of the total variance and where Pb contained the highest loading scores (0.62). Besides, PC3 explained 17% of the total variance and was dominated by Fe (0.55), whereas PC4 explained 10% of the total variance with a maximum loadings of Zn (0.56) and Hg (0.57). The loadings of Zn and Hg are very close in PC4, which represented a similar source of these metals. However, PC5 and PC6 explained around 7.82% and 6% of total variance with maximum loading of Zn (0.55) and Fe (0.67), respectively. Besides, PC7 explained 3% of the total variance with a maximum loading of Hg (0.51) and Pb (0.68), and PC8 explained 0.37% of the total variance with moderately favorable loading of Sr (0.66). From the component seven, the importance of Hg and Pb are very close, which reflects the precise origin of the metals. Although Pb occurs naturally in the environment, anthropogenic activities such as fossil fuel burning, mining, and manufacturing around the area contribute to the release of high concentrations [105]. Hg is utilized in the electrical industry (switches, thermostats, batteries) and other numerous industrial processes, including the production of caustic soda, in nuclear reactors, as antifungal agents for wood processing, as a solvent for reactive and precious metal, and as a preservative of pharmaceutical products [106].

**Figure 3:**
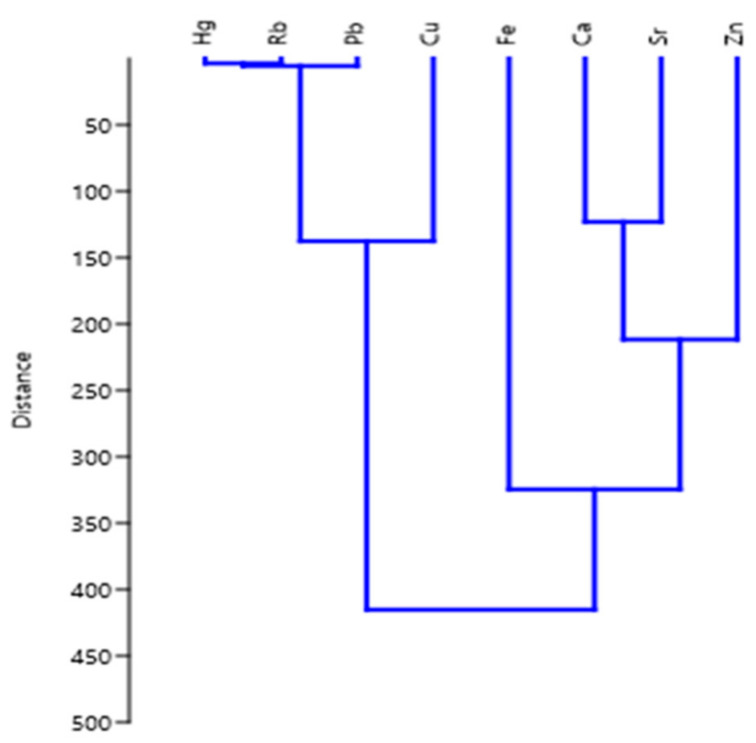
Hierarchical cluster analysis (dendrogram) of the variables (metals) in the study area.

Cluster analysis classifies variables into homogenous clusters in the form of dendrogram with variables that show similarities in the same group and dissimilarities between different groups [107]. Hierarchical cluster analysis (HCA) was used to determine the relationship between the metal concentration and their possible source. The HCA was established at (Dlink/Dmax) × 100 < 0.2, the Euclidean distance of similarities in variables, which represented two distinct groups of clusters (Figure 3). Cluster 1 consisted of Hg, Rb, Pb, and Cu that could have been arising from agricultural activities, electroplating materials and lubricants used near the study area. Besides, Fe, Ca, Sr, and Zn confined in cluster 2 that could have been attributed to natural (rock and soil weathering, etc.) or human activities like chemical and pharmaceutical industries, tanneries, industrial effluents, and others. Furthermore, correlations among the metals identified in the multivariate analyses also indicated the resemble accumulative characteristics for those metals presented in the aquatic organisms [108].

## 4. Conclusions

This study provides information on the levels of fourteen trace and heavy metals (Pb, Cr, Cu, Zn, Mn, Fe, Hg, Ni, Ca, Co, Se, Rb, Sr, and As) in the fifteen commercial fish species collected from the local fish market of Noakhali and the lower Meghna River. Iron (Fe) showed the highest accumulation level, whereas the Pb, Cr, Ni, and As levels in the muscle tissue of the studied fish species were lower than the permitted limits suggested by the WHO and FAO. However, the concentrations of Cu, Zn, Fe, Hg, and Ni in the fish muscle tissue exceeded the permissible limits suggested by the WHO and FAO. Among the organisms Tilapia was the most susceptible to metal accumulation and poses the maximum risks. Besides, the carnivorous, benthic, and the euryhaline species were the highest accumulator of metals on that area, which revealed the influence of habitat preferences and bio-magnification of metals through food cycle. EDI, THQ, and HI values for both adults and children were within the threshold limit and depicted that none experienced non-carcinogenic health risks. On the other hand, the carcinogenic health risks for Pb and Cr in all fish species were in the safe range (10^−6^ to 10^−4^) for adults and children. However, the correlation matrix, PCA, and hierarchical cluster dendrogram demonstrated that most of the elements in fishes arose from the anthropogenic sources. Hence, to protect the consumers from the derogative health risk effect, the release of toxic chemicals should be checked in a proper monitoring process.

## Figures and Tables

**Table 1:** Habitat, feeding habit, length & weight of fish samples collected from Noakhali fish market. (No. of samples, *n* = 2 for all species).

Common Name	Scientific Name	Habitat	Feeding Habit	Length (cm)	Weight (gm)
Tilapia	*Oreochromis mossambicus*	Freshwater	Omnivorous	23.75 ± 1.77	324.5 ± 4.95
Koi	*Anabas testudineus*	Freshwater	Carnivorous	16 ± 5.66	116.5 ± 94.04
Catla	*Gibelion catla*	Freshwater	Planktivorous	24.5 ± 1.41	578.5 ± 26.16
Rui	*Labeo rohita*	Freshwater	Herbivorous	33 ± 4.24	763.5 ± 23.33
Grass carp	*Ctenopharyngodon idella*	Freshwater	Herbivorous	31.5 ± 2.12	709 ± 41.01
Bighead carp	*Aristichthys nobilis*	Freshwater	Plankton and detritus feeder	32.5 ± 2.12	581 ± 36.06
Kalibaus	*Labeo calbasu*	Freshwater	Detritus feeder	32.5 ± 0.71	440.5 ± 6.36
Bata	*Cirrhinus reba*	Freshwater	Bottom feeder, herbivorous	22.5 ± 0.71	121 ± 1.41
Pabda	*Ompok pabda*	Freshwater	Omnivorous	13.5 ± 0.71	24.5 ± 4.95
Poa	*Otolothoides pama*	Freshwater, brackish, marine	Carnivorous	24.75 ± 1.06	192 ± 5.66
Chiring	*Apocryptes bato*	Freshwater, brackish, marine	Carnivorous	16 ± 1.41	21 ± 4.24
Ricksha	*Polynemus paradiseus*	Marine, freshwater, brackish	Feeds mainly on crustaceans (especially shrimps), small fishes, benthic organisms	13.25 ± 1.06	31 ± 11.31
Gulio	*Mystus gulio*	Brackish water	Carnivorous	15.75 ± 1.06	64 ± 5.66
Loitta	*Harpadon nehereus*	Marine, brackish	Carnivorous & to some extent cannibalistic	24.5 ± 2.12	118.5 ± 30.40
Koral	*Lates calcarifer*	Catadromous	Carnivorous	27.25 ±1.06	356.5 ± 12.02

**Table 2:** The mean heavy and trace metal concentration (mg/kg) in the tissues of the examined species from the Noakhali fish market. (No. of samples, *n* = 2 for all species).

Species		Pb	Cr	Cu	Zn	Mn	Fe	Hg	Ni	Ca	Co	Se	Rb	Sr	As
Tilapia	Mean	0.43	BDL	36.15	122.66	BDL	150.09	1.26	BDL	217.66	BDL	BDL	2.64	166.36	BDL
	SD	0.03		3.11	17.23		18.46	0.16		18.26			0.12	23.04	
Koi	Mean	0.68	7.86	32.49	107.22	7.10	146.82	0.98	BDL	80.23	BDL	7.64	1.72	33.27	BDL
	SD	0.06	0.54	3.73	11.21	3.03	0	0.24		70.23		0.54	0.17	15.81	
Catla	Mean	0.57	BDL	33.92	105.05	BDL	168.76	2.58	BDL	29.21	BDL	BDL	3.29	14.06	BDL
	SD	0.27		1.19	1.06		7.43	0.15		4.23			0.18	3.18	
Rui	Mean	0.60	BDL	32.49	131.42	BDL	147.55	1.57	BDL	118.34	BDL	BDL	1.31	69.53	BDL
	SD	0.10		0.40	2.95		5.64	0.13		23.83			0.43	7.23	
Grass carp	Mean	0.53	7.69	48.59	116.82	6.91	204.83	1.38	BDL	44.00	BDL	9.75	3.60	31.65	BDL
	SD	0.05	2.10	9.94	9.20	2.23	27.69	0.12		1.89		1.71	0.96	8.50	
Bighead carp	Mean	0.61	5.90	33.32	102.06	BDL	208.51	1.33	BDL	68.34	BDL	BDL	2.47	35.72	BDL
	SD	0.28	1.74	2.55	4.15		13.42	0.01		17.68			0.27	5.20	
Kalibaus	Mean	0.54	BDL	37.76	108.22	BDL	149.18	0.72	BDL	50.34	BDL	9.52	1.45	27.20	BDL
	SD	0.21		9.94	8.14		0.77	0.47		5.09		0.21	0.16	5.96	
Bata	Mean	0.63	5.99	35.56	144.35	BDL	156.61	1.74	1.04	76.90	BDL	9.15	1.23	58.38	BDL
	SD	0.10	0.18	10.55	5.20		26.15	0.55	0.08	10.95		0.01	0.14	2.77	
Pabda	Mean	0.53	BDL	31.63	101.88	5.485	148.63	0.79	1.55	73.84	BDL	BDL	2.09	31.862	BDL
	SD	0.27		1.27	6.73	0	12.82	0.15	0.46	12.20			0.003	5.43	
Poa	Mean	0.38	9.69	30.29	107.22	BDL	177.46	2.06	BDL	108.62	BDL	9.27	1.77	153.69	BDL
	SD	0.12	2.04	3.93	16.87		15.12	0.13		12.78		1.77	0.21	6.41	
Chiring	Mean	0.65	7.35	46.98	111.81	BDL	151.17	1.21	BDL	127.10	4.70	8.20	1.24	135.29	BDL
	SD	0.03	3.18	24.22	6.37		1.02	0.03		3.75	2.55	1.10	0.04	74.17	
Ricksha	Mean	0.48	7.24	33.95	101.38	BDL	188.15	2.03	BDL	79.21	BDL	8.85	1.52	96.41	BDL
	SD	0.07	3.59	4.14	8.38		29.23	0.37		54.77		0.06	0.15	55.59	
Gulia	Mean	0.57	BDL	46.57	129.33	BDL	150.81	1.42	BDL	116.30	2.89	BDL	1.16	103.85	BDL
	SD	0.04		12.39	13.45		3.59	0.25		39.20	0.84		0.09	42.14	
Loitta	Mean	0.20	BDL	35.40	106.72	BDL	148.82	2.90	BDL	76.02	BDL	8.12	1.51	111.32	BDL
	SD	0.02		17.83	6.02		12.56	0.37		21.23		1.94	0.15	20.19	
Koral	Mean	0.44	8.84	31.44	103.72	BDL	135.58	2.33	BDL	51.29	BDL	9.39	1.69	58.48	BDL
	SD	0.05	0.12	2.97	13.81		9.23	0.24		49.86		1.94	0.11	43.30	

BDL = Below Detection Limit.

**Table 3:** Comparison of metals in fishes from Noakhali fish market with different international guidelines and other studies in the world (in mg/kg dry weight).

Standards	Cu	Pb	Ni	Hg	Fe	Zn	Cr	References
Noakhali fish market	36.44	0.521	1.467	1.657	162.198	113.326	7.336	This study
FAO	30	2	55	0.5	180			[62]
WHO	30	0.5	30	0.5	109			[63]
ROPME	0.5–19.5	0.01–1.28	0.01–0.75	1	200			[64]
FDA		1.7	70	0.5–1				[65]
European Commission		1	40	0.5–1				[66]
NOAA	149	128	52	0.5	250			[67]
FAO/WHO limits	30			0.5	333.3	100		[68]
FSG	30	2	80			30	12–13	[62,69]
Bangladesh	5	0.3					1	[70]
India	30	0.3		0.5		50		[71]
Malaysia	30	0.3				100		[72]
China	50	2		0.3				[73]
International criterion	15	0.3		0.5		60		[74]
Bangshi river, Bangladesh	22.8	4.64	2.59			168.97	1.12	[32]
Meghna river estuary, Bangladesh	4.97	3.66					0.76	[75]
Dhaleshwari river, Bangladesh	5.17–7.48	4.25–8.17						[29]
Gorgan Bay, Iran		0.43			501.65		6.4	[76]
Asafo market, Ghana	0.02–0.156	0.054–0.085				0.016–0.022		[59]
Pearl river, China	1.17–6.72	0.05–1.94				2.62–20.2		[60]

**Table 4:** The Metal Pollution Index (MPI) of the examined species from the Noakhali fish market.

Species	Metal Pollution Index (MPI)
Tilapia	20.73166
Koi	11.87546
Catla	13.63232
Rui	16.83319
Grass carp	13.24696
Bighead carp	13.98457
Kalibaus	11.80463
Bata	10.97328
Pabda	9.818907
Poa	16.12621
Chiring	11.47850
Ricksha	14.69736
Gulia	14.56895
Loitta	14.80534
Koral	13.41669

**Table 7:** Estimated Carcinogenic Risk of metals detected in the targeted fish species of Noakhali fish market.

Species	Carcinogenic Risk (Pb)		Carcinogenic Risk (Cr)	
	Csf: 0.0085 *		Csf: 0.5 **	
	Adult	Child	Adult	Child
Tilapia	2.864 × 10^−9^	1.264 × 10^−8^	8.629 × 10^−5^	3.809 × 10^−4^
Koi	4.583 × 10^−9^	2.023 × 10^−8^	3.115 × 10^−6^	1.375 × 10^−5^
Catla	3.838 × 10^−9^	1.694 × 10^−8^	-	-
Rui	4.074 × 10^−9^	1.798 × 10^−8^	-	-
Grass carp	3.572 × 10^−9^	1.577 × 10^−8^	3.048 × 10^−6^	1.346 × 10^−5^
Bighead carp	4.111 × 10^−9^	1.815 × 10^−8^	2.341 × 10^−6^	1.033 × 10^−5^
Kalibaus	3.609 × 10^−9^	1.593 × 10^−8^	-	-
Bata	4.266 × 10^−9^	1.883 × 10^−8^	2.374 × 10^−6^	1.048 × 10^−5^
Pabda	3.569 × 10^−9^	1.575 × 10^−8^	-	-
Poa	2.547 × 10^−9^	1.125 × 10^−8^	3.839 × 10^−6^	1.695 × 10^−5^
Chiring	4.367 × 10^−9^	1.928 × 10^−8^	2.913 × 10^−6^	1.286 × 10^−5^
Ricksha	3.238 × 10^−9^	1.429 × 10^−8^	2.868 × 10^−6^	1.266 × 10^−5^
Gulia	3.828 × 10^−9^	1.690 × 10^−8^	-	-
Loitta	1.362 × 10^−9^	6.009 × 10^−9^	-	-
Koral	2.949 × 10^−9^	1.302 × 10^−8^	3.503 × 10^−6^	1.545 × 10^−5^
Mean	2.932 × 10^−9^	1.553 × 10^−8^	1.225 × 10^−5^	5.409 × 10^−5^

* [49] ** [100].

**Table 8:** Pearson correlation matrix of the metals in fish samples collected from the study area.

	Cu	Ca	Fe	Zn	Hg	Pb	Rb	Sr
Cu	1							
Ca	0.09467	1						
Fe	0.15372	−0.26686	1					
Zn	0.30966	0.3802	−0.2181	1				
Hg	−0.2560	−0.23051	0.0389	−0.1159	1			
Pb	0.21208	−0.09375	0.0699	0.28540	−0.6004 *	1		
Rb	0.09426	−0.20642	0.555 *	−0.2621	0.05301	−0.0289	1	
Sr	0.12102	0.8033 **	−0.1565	0.17546	0.185543	−0.4541	−0.3205	1

* *p* < 0.05, ** *p* < 0.01.

**Table 9:** Component matrix of eight factors model with moderate loadings in fish.

Metals	PC 1	PC 2	PC 3	PC 4	PC 5	PC 6	PC 7	PC 8
Cu	0.077094	0.35666	0.48445	0.40975	−0.63966	−0.14283	0.1046	−0.15416
Ca	0.53915 *	0.048427	0.27734	−0.38842	0.25127	−0.11289	0.14619	−0.61801
Fe	−0.37036	0.0087578	0.55052 *	−0.05238	0.1909	0.67073 *	−0.22546	−0.14067
Zn	0.33914	0.33803	0.052466	0.56126 *	0.5497 *	−0.06207	−0.35525	0.14394
Hg	−0.03564	−0.56239	0.028292	0.56628 *	0.18927	0.078451	0.51397 *	−0.23416
Pb	−0.1196	0.61867 *	−0.15557	−0.05853	0.20548	0.24232	0.67972 *	0.11118
Rb	−0.4017	−0.032157	0.4909	−0.13855	0.32422	−0.6287	0.14656	0.23574
Sr	0.52369 *	−0.23672	0.34145	−0.14206	−0.07699	0.23022	0.20734	0.65652 *
Eigenvalue	2.40605	1.99291	1.36017	0.856832	0.625241	0.488137	0.241364	0.0292975
% variance	30.076	24.911	17.002	10.71	7.8155	6.1017	3.0171	0.36622
Cumulative Variance %	30.076	54.987	71.989	82.699	90.5145	96.6162	99.6333	99.999

Extraction Method: Principal Component Analysis (PAST), * Moderate loading value (>0.5).

## Data Availability

Data are provided in the article.
